# Visualizing the Effect of Process Pause on Virus Entrapment During Constant Flux Virus Filtration

**DOI:** 10.3390/membranes16010006

**Published:** 2025-12-26

**Authors:** Wenbo Xu, Xianghong Qian, Hironobu Shirataki, Daniel Straus, Sumith Ranil Wickramasinghe

**Affiliations:** 1 Department of Biomedical Engineering, University of Arkansas, 1475 West Cato Springs Road, Fayetteville, AR 72701, USA; wenboxu@uark.edu; 2 Bioprocess Division, Asahi Kasei Life Science Corporation, Hibiya Mitsui Tower, 1-1-2 Yurakucho, Chiyoda-ku, Tokyo 100-0006, Japan; shirataki.hb@om.asahi-kasei.co.jp; 3 Purification Research and Development, Asahi Kasei Bioprocess America, Inc., 1855 Elmdale Ave, Glenview, IL 60026, USA; daniel.strauss@ak-bio.com; 4 Ralph E. Martin Department of Chemical Engineering, University of Arkansas, 1475 West Cato Springs Road, Fayetteville, AR 72701, USA

**Keywords:** aggregation, flux, hollow fiber, laser scanning confocal microscopy, log removal of virus, minute virus of mice, monoclonal antibody, polymeric membrane, process interruption, virus clearance

## Abstract

Virus filtration is an essential unit operation used to validate clearance of adventitious virus during the manufacture of biopharmaceutical products such as monoclonal antibodies. Obtaining at least a 10,000-fold reduction in virus particles in the permeate is challenging as monoclonal antibodies are about half the size of the virus particles. Minute virus of mice, FDA-recommended model adventitious virus, was labeled with a fluorescent dye. Laser scanning confocal microscopy was used to determine the location of virus entrapment within the virus filtration membrane. Three different hollow fiber membranes made of regenerated cellulose and polyvinylidene fluoride were tested. Feed streams consisted of MVM spiked in buffer and MVM spiked in 5 g L^−1^ bovine serum albumin known to contain aggregates similar in size to the MVM. After filtering the feed, a buffer flush was used, with and without 30 min pause before the buffer flush. For all virus filters, a 30 min process pause led to broadening and movement of the virus entrapment zone deeper into the membrane. The presence of aggregates led to greater broadening of the entrapment zone. Both effects could lead to reduced virus clearance. Visualization of virus entrapment helps improve understanding of the behavior of virus filtration membranes.

## 1. Introduction

The term virus filtration is frequently used in the biopharmaceutical industry. It generally refers to a membrane-based unit operation designed to validate clearance of contaminating virus particles from a biotherapeutic product [1]. This pressure-driven membrane separation process is somewhat similar to ultrafiltration [2]. The most commonly used virus filters are designed to ensure the biotherapeutic is not contaminated by retrovirus and adventitious virus particles [3]. Typically, model virus particles such as minute virus of mice (MVM), an FDA-approved model parvovirus, is spiked into a feed stream containing the biotherapeutic of interest. Scaled down testing is conducted in order to determine the level of virus clearance [4].

Unlike ultrafiltration, virus filtration is routinely run in normal flow mode. Further, the support structure of these asymmetric membranes faces the feed stream and acts as an inline prefilter, removing contaminants that could foul the membrane barrier layer [5]. Virus filters are single-use devices. The performance requirements for virus filtration are far more challenging than for ultrafiltration. Often the biotherapeutic, e.g., a monoclonal antibody (mAb), has a hydrodynamic diameter of around 10–12 nm [6]. About 95% or more of the biotherapeutic must be recovered in the permeate. MVM on the other hand is a non-enveloped virus with the capsid being about 20–25 nm in diameter [7]. This FDA-recommended model virus, for adventitious virus clearance, is frequently used in virus spiking studies to validate virus clearance. However, at least a 10,000 fold reduction or 4 log removal value (LRV) in the permeate is required for effective virus clearance. Requirements for membrane throughput (product recovered per membrane surface area) and permeate flux are also challenging [8].

Virus filtration occurs towards the end of the purification train; thus, fouling is usually due to product-related foulants such as irreversible and reversible aggregates, misfolded proteins, etc. Further, given the 2-fold difference in size between the virus particles to be rejected and the biotherapeutic to be recovered in the permeate, and the fact that the membrane has a pore size distribution less than 4 LRV, fouling can occur depending on feed and operating conditions [9]. In particular, flow interruption followed by process pause can lead to reduced levels of virus clearance.

Virus filtration is usually run at constant pressure. Process interruptions or depressurization of the feed stream can occur during switching of the feed from one tank to another if multiple feed tanks are used. In addition, a buffer flush is often used at the end of the filtration to recover product in the system hold up volume. When the feed is switched to the buffer, a process interruption may occur [10].

Several previous studies have shown that a process interruption can lead to reduced LRV [11,12]. In our recent work [13] we show that operating virus filters at very low permeate flux (5 LMH), and therefore low pressure for a low-fouling monoclonal antibody solution spiked with MVM, can lead to significant virus breakthrough and much lower LRVs for many commercial virus filters investigated. When operated at pressures recommended by the manufacturers where convection dominates, very little or no virus breakthrough was observed for all these virus filters. We adopted a Peclet number to describe the relative contribution of diffusion and convection to virus particle migration. When the Peclet number is ≪ 1, and diffusion of virus particles becomes significant, virus breakthrough and reduced LRVs can occur. However, the Peclet number at which diffusion dominates over convection will depend on membrane morphology and is likely to be different for membranes from different manufacturers. During process interruption, the pressure drops to zero, and diffusion of virus particles to larger pores in the membrane can lead to virus particles passing into the permeate.

Wood and Zydney [14] studied the effect of pressure release that occurs during a process interruption on retention of a bacteriophage by a relatively symmetric membrane. In these studies, the concentration of bacteriophage in the permeate increased following a pressure release. The results were described by an internal polarization model [15]. LaCasse et al. [16] investigated a number of different pressure release steps including rapid pressure reduction, multistep pressure reduction, high pressure to low pressure transition, as well as a process pause. In general, when the filter is operated under conditions where virus diffusion could be significant, a lower LRV is often observed. In those studies, only the titer of virus particles in the permeate was determined.

In a more recent study, Afzal and Zydney (2022) [17] investigated the effect of both permeate flux and process interruption on virus retention for a relatively symmetric virus filtration membrane. A bacteriophage was used, and the titer in the permeate was determined at a range of fluxes and after a process interruption. The results confirmed our previous findings that at low fluxes, when diffusion of virus particles is significant, as well as after a process interruption when the pressure drops to zero, the LRV decreases. Peles et al. [18] extend this work, suggesting that at low fluxes when virus particles can diffuse out of pore constrictions and into larger pores, a lower LRV is observed. Further process interruptions can lead to a much greater reduction in LRV when the pressure drops to zero and convective flow ceases for a brief period.

Our most recent work [19] on visualizing entrapped fluorescently labeled MVM particles during virus filtration using three different commercial virus filters found that the entrapment zone of MVM particles can migrate deeper into the filters at lower flux and longer operation times. This occurred for three different feed streams containing buffer only, bovine serum albumin (BSA), and mAb. This again suggests that diffusion can also play an important role even at flux ranges when the Peclet number is more than 1. Moreover, the presence of protein or protein aggregates can impact the entrapment zone of the virus particles due to pore constriction and competition for entrapment sites.

The aim of this work was to visualize the location of virus entrapment during virus filtration with process interruption and process pause. Three different commercially available hollow-fiber virus filters were investigated. MVM was labeled with DyLight™ 633 NHS Ester Fluorescent dye. Laser scanning confocal microscopy (LSCM) was used to locate captured virus particles within the membrane. Two feed streams were investigated: PBS buffer and 5 g L^−1^ BSA in the same buffer that forms low-molecular-weight aggregates. Both feed streams were spiked with MVM. All three virus filters were challenged with 1000 L m^−2^ of feed solution followed by 100 L m^−2^ buffer flush. Switching to the buffer flush resulted in a process interruption. In addition, the effect of a 30 min process pause, after process interruption, on the location of MVM entrapment was also investigated for the three hollow-fiber virus filters.

Leisi et al. [20] indicate that while bacteriophage models and nanoparticles provide general insights into virus retention in virus filtration membranes, often significant differences are observed when compared to FDA-recommended model viruses such as MVM particles [21,22]. In this work the focus was to determine the location of virus entrapment under industrially relevant conditions. Therefore, fluorescently labeled MVM was used to determine the location of virus entrapment. Further, all experiments were conducted at constant flux at 75 L m^−2^ h^−1^ for a total of 12 h of filtration time. A decrease in permeate flux can lead to virus diffusion to larger pores. Under constant pressure operation, the permeate flux could decrease with throughput, leading to a change in the importance of diffusion versus convection of virus particles. To overcome this complication, experiments were conducted at constant flux. Changes in the location of virus entrapment will be due to the presence of BSA (and aggregates) as well as diffusion during the process pause.

## 2. Materials and Methods

### 2.1. Materials and Chemicals

BSA was purchased from Lee BioSolutions (Maryland Heights, MO, USA). The following chemicals (biotechnology grade) were used to produce the PBS buffer solution: potassium chloride obtained from VWR life Science (Avantor, Radnor, PA, USA), and sodium phosphate dibasic, sodium chloride, and potassium dihydrogen phosphate that were purchased from MilliporeSigma (Burlington, MA, USA). Biotechnology grade tris base and ethylenediaminetetraacetic acid (EDTA) used to prepare the TNE buffer were obtained from MilliporeSigma. MVM virus stock and A9 mouse fibroblast cells (ATCC^®^ CCL-1.4™) were obtained from the American Type Culture Collection (Manassas, VA, USA). Glucose-rich and advanced DMEM media were purchased from MilliporeSigma. Fetal bovine serum (FBS), DyLight™ 633 NHS Ester dye, and 4% paraformaldehyde (PFA) in PBS were obtained from Thermo Fisher (Waltham, MA, USA). Tissue-Tek O.C.T. Compound (Sakura Finetek, Torrance, CA, USA) was used for cryostat sectioning, and deionized water (DI water) was prepared using a Thermo Scientific Barnstead Smart2Pure UV/UF system (Waltham, MA, USA).

Planova™ S20N, Planova™ 20N, and Planova™ BioEX virus filters were provided by Asahi Kasei Life Science Corporation (Tokyo, Japan). Table 1 gives detail of the virus filters. The feed throughput was 1000 L m^−2^ followed by 100 L m^−2^ buffer flush.

### 2.2. Cell Culture

Table 2 summarizes the experiments conducted using the three virus filters investigated here. All experiments included a buffer flush (100 L m^−2^). This involved a process interruption when the feed stream to the virus filter was switched to buffer at the end of the run. This was performed in two ways: immediately (without process pause) and after a 30 min delay (with process pause).

PBS was prepared by combining 137 mmol L^−1^ NaCl, 8.1 mmol L^−1^ Na_2_HPO_4_, 2.7 mmol L^−1^ KCl, and 1.5 mmol L^−1^ KH_2_PO_4_ in DI water. The DI water and buffer, after preparation, were filtered through a 0.22 µm PES bottle top filter. The pH was adjusted to 7.36 with 2 mol L^−1^ phosphoric acid. BSA solutions (5 g L^−1^) were prepared in the PBS buffer. After preparation of the BSA solution, it was filtered through a 0.1 µm PES bottle top filter (Thermo Fisher, Waltham, MA, USA). BSA concentrations were determined by A_280_ measurement using a Genesys 100UV spectrophotometer (Genesys 100UV scanning system, Thermo Fisher).

The aggregates in the BSA feed solution were determined using an Agilent Infinity 1260 Series HPLC system (Agilent, Santa Clara, CA, USA) equipped with a size exclusion chromatography (SEC) column (TSK gel G3000 SWxl, 300 mm × 7.8 mm, MilliporeSigma). The mobile phase was pH 7.36 PBS buffer run at a flow rate of 1.0 mL min^−1^. BSA aggregates were detected by UV absorbance at 220 nm.

**Figure 1:**
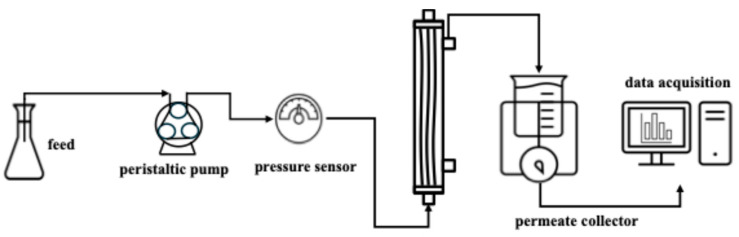
Schematic diagram of constant flux filtration setup.

Prior to virus filtration a visual leak test (VLT) and equilibration was performed according to the manufacturer’s protocol [23]. The experimental setup for constant flux filtration conducted here is shown in Figure 1. A MasterFlex L/S digital pump (Cole-Parmer, VWR, PA, USA) was used to control the flux. Experiments with Planova™ S20N, 20N, and BioEX virus filters were performed at a constant flux of 75 L m^−2^ h^−1^ using PBS feed streams spiked with ~7.5 logCopies mL^−1^ labeled MVM (qPCR). In addition, for the S20N and 20N virus fitters, 5 g L^−1^ BSA was added to the same MVM containing feed stream. Both feed streams, MVM in buffer and MVM in 5 g L^−1^ BSA, were filtered using a 0.22 μm bottle top filter just prior to virus filtration (Thermo Fisher).

The target feed throughput was 1000 L m^−2^. In addition, 100 L m^−2^ buffer was used to flush the system. Consequently, every run included a process interruption when the feed was switched to the buffer flush. To evaluate the impact of a process pause after a process interruption, experiments were also conducted with a 30 min process pause prior to flushing the system with buffer. The buffer was also run through the virus filter at 75 L m^−2^ h^−1^.

The effect of labeling MVM on the pressure during constant flux filtration was determined by conducting additional experiments using the S20N filter with non-labeled MVM. Feed streams consisting of MVM spiked in PBS buffer and spiked in 5 g L^−1^ BSA in PBS were investigated.

The filtrate was collected in approximately 250 mL fractions and one buffer flush fraction. Filtration performance was monitored using a Mettler Toledo scale (Mettler Toledo, Columbus, OH, USA) connected to Balance Link software, with data recorded every minute and averaged over 10 min intervals.

### 2.3. MVM Production and Titer Determination

The method used to produce and purify MVM has been described by Asher et al. [24] and modified by Fan et al. [13]. A9 cells were cultured in T175 flasks (Thermo Fisher) with glucose rich DMEM supplemented with 10% FBS. When 90% confluence was reached, the cells were infected with MVM and incubated at 37 °C with 5% CO_2_ in 40 mL advanced DMEM containing 1% FBS. The MVM particles were harvested 21 h after inoculation, clarified by centrifugation, and sterile filtered using a 0.22 µm bottle top filter (Thermo Fisher). The virus was concentrated and buffer-exchanged into TNE buffer (10 mM Tris, 150 mM NaCl, 1 mM EDTA) with 100 kDa Amicon Ultra-15 centrifugal filters (MilliporeSigma). The virus was purified by ultracentrifugation (Beckman Coulter, Brea, CA, USA). MVM titers ranged from 10.5 to 11.5 logCopies mL^−1^ (determined by qPCR). MVM was spiked into feed solutions to a final titer of ~7.5 logCopies mL^−1^. MVM titers were determined using quantitative PCR (qPCR) and infectivity assays. The qPCR method used has been described by Chen et al. [25]. qPCR was used to estimate feed titers (~7.5 logCopies mL^−1^). It detects both infectious and noninfectious particles. Infectivity was measured by TCID_50_ assay using NB324K cells (donated by Peter Tattersall, Yale University, New Haven, CT, USA) following the method described by Fan et al. [13]. The titers were calculated by the Spearman–Kärber method [26] with 95% confidence limits. When TCID_50_ titers were below detection, large volume plating (LVP) was used for increased sensitivity [13].

### 2.4. MVM Labeling

MVM particles were fluorescently labeled with DyLight™ 633 NHS Ester dye prior to spiking as described in our recent work [19]. Briefly, 1.2 mL of purified MVM stock (11.10 logCopies mL^−1^, qPCR) were buffer-exchanged into the labeling buffer (0.1 M sodium phosphate, 0.15 M NaCl, pH 7.36) using a 100 kDa Amicon Ultra-15 centrifugal filter using 50–100 diavolumes. The virus was then incubated with dye (concentration at 1 g L^−1^) for 1 h under gentle agitation. Labeled particles were washed with 100 diavolumes of labeling buffer using the same filter to remove unbound excess dye. When using labeled MVM, the virus filtration setup was shielded from light with aluminum foil to minimize photobleaching.

### 2.5. MVM Detection Using Laser Scanning Confocal Microscopy (LSCM)

The procedures for preparing fibers and LSCM imaging were detailed in our earlier research [19]. Following virus filtration, the hollow fibers were removed from the module and fixed overnight in 4% PFA to immobilize the virus particles. The fibers were then rinsed with PBS, sectioned into small pieces, and embedded in Tissue-Tek O.C.T. Compound at −20 °C for cryosectioning. Cross-sections (8 µm) were prepared using a Leica CM 1800 cryostat (Leica Biosystems, Buffalo Grove, IL, USA). The sliced fiber was mounted on a glass slide and sealed with a coverslip using nail polish. Imaging was performed on a Leica TCS SP5 LSCM (Leica, Buffalo Grove, IL, USA) at 10× and 40× magnifications. DyLight™ 633 NHS Ester was excited with a 638 nm laser. Emissions between 640 and 670 nm were collected. Brightfield (white light), fluorescence, and overlapped images were obtained using LAS X software 3.7.0 (Leica) and analyzed with ImageJ (ver. Fuji, Wayne Rasband, NIH, Bethesda, MD, USA). Brightfield images were used to define fiber inner (feed) and outer (permeate) surfaces, and fluorescence intensities were normalized to 100 and plotted against relative membrane depth (inner surface = 0, outer surface = 1).

## 3. Results

### 3.1. Permeate Flux

**Figure 2:**
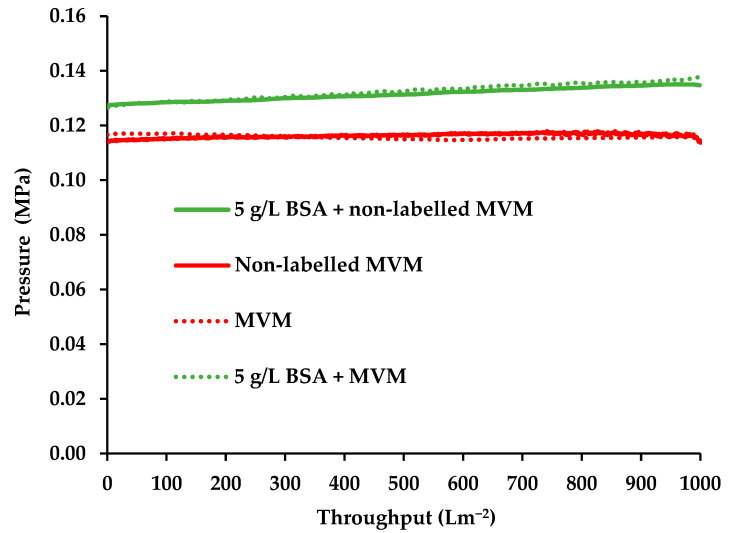
Variation in pressure with throughput for S20N.

A number of control experiments were conducted using the S20N filter in order to determine the effect of labeled and non-labeled MVM on the pressure during virus filtration. Figure 2 gives the variation in pressure with throughput for PBS buffer spiked with labeled and non-labeled MVM. The variation in pressure with throughput is also given for feed streams containing 5 g L^−1^ BSA spiked with labeled and non-labeled MVM.

The results in Figure 2 indicate that the variation in pressure with throughput for labeled and non-labeled MVM is almost the same. Labeling MVM had no effect on the permeate pressure, and consequently all subsequent experiments were conducted with labeled MVM only. The increase in pressure with throughput for the buffer streams spiked with MVM is minimal, indicating there is very little fouling. This confirms the purity of the MVM spike.

The pressure versus throughput curves for the feed streams containing BSA lie above the curves for buffer. Further, the increase in pressure with throughput is slightly greater than for buffer. This indicates that the resistance to permeate flow is greater for the feed streams containing BSA. Table 3 gives SEC results for the BSA. As can been seen, the BSA does aggregate, leading to the presence of about 13% dimers and 2% trimers. Irreversible aggregation of BSA occurs through an intermolecular thiol-disulfide interchange reaction [27]. However, the occurrence of this process and the degree of dimerization, trimerization, etc., depends on the properties of the BSA sample, which vary among suppliers. These aggregates as well as BSA could adsorb in the pores of the membrane, leading to initial pore constriction and blocking, as well as a slowing, the cake formation process [28]. This would explain the higher pressure required for a flux of 75 L m^−2^ h^−1^ when compared to buffer-only condition. In addition, the viscosity of the BSA containing feed streams will be slightly higher, resulting in a higher pressure for a given permeate flux.

The flux chosen to compare the performance of the three virus filters was 75 L m^−2^ h^−1^ based on the manufacturer’s recommended operating conditions given in Table 1. The 20N has the lowest maximum recommended operating pressure specified by the manufacturer. Figure 2 indicates that a flux of 75 L m^−2^ h^−1^ results in a pressure above the maximum recommended operating pressure. The S20N and BioEX have higher maximum recommended operating pressures specified by the manufacturer. As shown in our previous work [13], if virus filters are run at low fluxes, a reduced LRV can result. Thus, the highest flux that could be used for all three virus filters was chosen in order to ensure the results are of industrial relevance.

The results in Figure 2 do not include the variation in pressure during the buffer flush. No significant change in pressure was observed with and without process pause for the buffer flush. Further, the pressure was about the same as for the feed stream before buffer flush.

**Figure 3:**
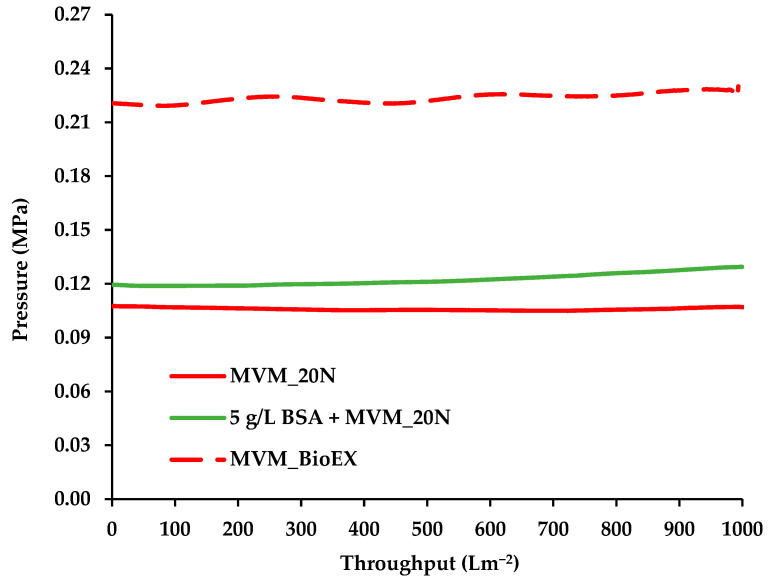
Variation in pressure with throughput for 20N and BioEX.

The variation in pressure with throughout for the 20N virus filter is given in Figure 3. Results are given for buffer spiked with labeled MVM and buffer containing 5 g L^−1^ BSA spiked with labeled MVM. The results are analogous to Figure 2; the feed stream containing BSA exhibits a slightly higher pressure. The S20N and 20N virus filters both contain regenerated hollow fibers. The S20N is a newer version of the 20N. As indicated in Table 1, the filter operates at a higher pressure than the 20N. The resistance to permeate flow for the S20N is a little higher than the 20N, hence the pressure is slightly higher for all feed streams.

In order to investigate the effect of polymer properties on permeate flux, the BioEX virus filter which contains PVDF hollow fibers was tested with a feed stream containing buffer spiked with MVM only. The result is also given in Figure 3. While the pressure profile is similar to those obtained for the S20N and 20N, the pressure is much higher. Table 1 indicates that the BioEX operates at a much higher pressure than either the S20N or 20N. Consequently, the pressure for a permeate flux of 75 Lm^−2^h^−1^ is much higher due to the higher resistance to permeate flow through membrane. All the pressure profiles show a slight increase in pressure with throughput. However, the level of membrane fouling appears to be very low.

### 3.2. Visualization of Virus Capture

**Figure 4:**
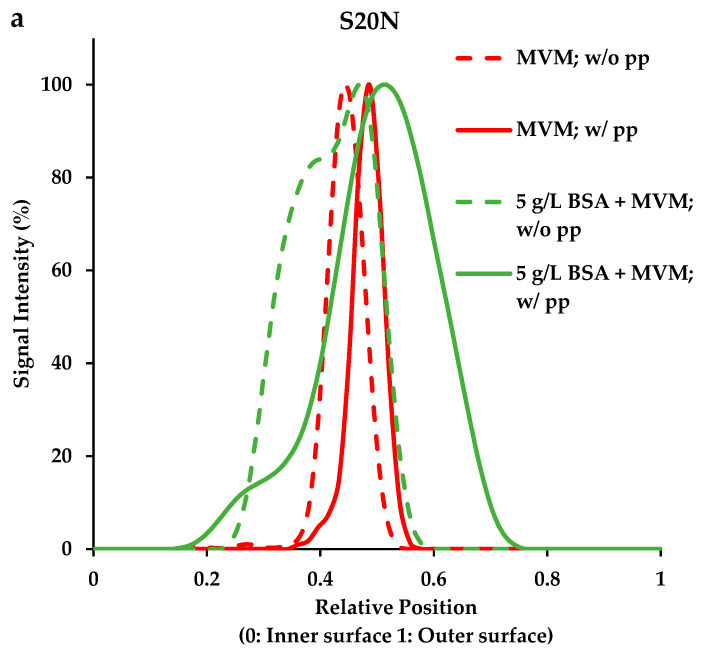

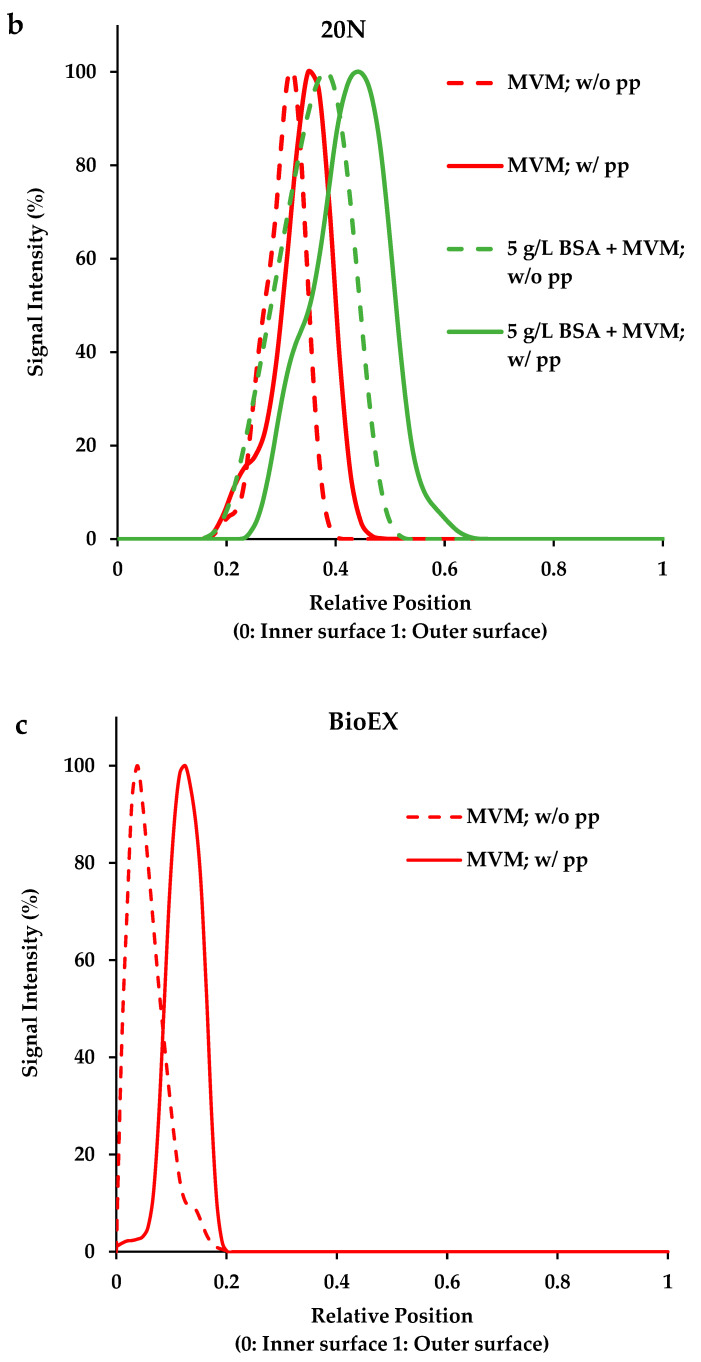
Location of MVM entrapment with and without process pause by LSCM. (**a**) Feed streams with and without 5 g L^−1^ BSA using S20N, (**b**) feed streams with and without 5 g L^−1^ BSA using 20N, (**c**) feed streams with spiked MVM using BioEX. w/o = without process pause. w/pp = with process pause.

Figure 4a–c give capture profiles for the S20N, 20N, and the BioEX virus filters. The original LSCM images are given in Appendix A. The intensity profiles shown in Figure 4 were obtained via z-stack of confocal images by taking multiple focal planes along the z-axis across the fiber from the outside lumen surface of the hollow fiber to the inner lumen surface. Thus, they represent 3D imaging. The examples of the original LSCM images given in Appendix A represent an overlay of the fluorescent image at a given focal plane with a brightfield image of the cross-sectional area of the fiber without moving the sample. They represent a 2D image.

The results for all three filters indicate the following trends. A 30 min process pause associated with a process interruption for buffer flush leads to a migration of the virus entrapment zone deeper into the membrane. Some broadening of the entrapment zone can be observed. The presence of 5 g L^−1^ BSA which contains aggregates similar in size to the MVM particles leads to significantly more broadening of the entrapment zone within the membrane. The results for the S20N and 20N are similar. The membranes used in these two virus filters are similar, both being regenerated cellulose. However, the BioEX membrane is PVDF. As can be seen, virus particles are captured much closer to the feed side of the membrane. These results highlight the importance of membrane properties (morphology and surface properties) in determining the location of virus capture. Since virus filtration uses size exclusion to reject virus particles, membrane morphological properties such as pore size, pore size distribution, and porosity directly affect the observed permeate flux during constant pressure virus filtration and the location of virus capture. Membrane surface properties on the other hand will affect the location of virus capture due to their interaction with the product species or product variants (misfolded protein, product dimers, trimers, etc.).

### 3.3. Virus Clearance

Table 4 gives the results for virus clearance for feed streams spiked with labeled MVM for all three virus filters. Table 5 gives results for feed streams contain 5 g L^−1^ BSA. As can be seen at a flux of 75 L m^−2^ h^−1^, Table 4 and Table 5 indicate more than 4.5 LRV is obtained. In our earlier work we show that labeling MVM has no effect on the observed LRV [19]. Further, for the S20N and BioEX virus filters, no MVM particles were detected in all permeate fractions with LVP. Consequently, virus clearance was above level of detection. It is not possible to state which filter provides higher absolute LRV under these operating conditions. The 20N virus filter the LRV is also well above 4 and above the level of detection with the TCID_50_ assay, though not the LVP assay. BSA recovery was more than 99% for all feed streams containing BSA. The original data are given in Appendix A.

The results in Figure 4a–c and Table 4 and Table 5 provide visual evidence of the effects of process pause and aggregates on the zone of virus entrapment. As can be seen, a 30 min process pause leads to migration of virus particle deeper into the membrane with some peak broadening. It is likely that during a process pause, in the absence of an applied pressure, the virus particles can back diffuse and migrate into larger pores and are captured deeper within the membrane during the buffer flush.

These results are in agreement with previous studies using fluorescent nanoparticles [29] and bacteriophage [14,17,18] which indicate that a process pause can lead to reduced LRV. In the studies conducted here, the virus filters were run under industrially relevant conditions. The aim was not to overload the virus filters in order to observe changes in LRV after a process pause but rather observe changes in the location of virus capture.

The presence of BSA aggregates (dimers and trimers) leads to spreading of the entrapment zone. Figure 4a,b indicate that while the entrapment zone profiles are similar, the entrapment zone is larger for the S20N compared to the 20N. The 20N was tested at a pressure greater than the maximum recommended pressure by the manufacturer. The S20N was operated at about half the maximum recommended pressure. This result suggests that peak spreading does not depend on the maximum pressure recommendation for the filter.

The BioEX virus filter membrane is made from PVDF. Comparing Figure 4a,b with Figure 4c indicates that the capture profile for MVM spiked in buffer for BioEX is different than the profiles for S20N and 20N. While a 30 min process pause leads to movement of the virus capture zone deeper into the membrane, virus capture occurs much closer to the feed side of the membrane compared to the S20N and 20N. This is due to differences in membrane morphology (pore size, pore size distribution, and porosity. The results highlight the fact that the effects of aggregates of similar size to the virus particles and process pause also depend on membrane properties.

In this study the effect of the length of the process pause and multiple process pauses was not investigated. Earlier studies [17,18] using different membranes under different operating conditions, with bacteriophage as the model virus, provide some insights. Longer process pauses could lead to broadening and further migration of the entrapment zone deeper into the membrane. A similar effect is likely for multiple process pauses. Here a process pauses of 30 min was selected as longer process pauses are rare in practice.

The presence of BSA aggregates leads to broadening of the capture zone for all three filters. Previous investigators have shown that protein fouling (including model proteins like BSA) can lead to a decrease in LRV [30]. On the other hand, other studies have shown no effect of fouling, which leads to decreased fluxes during constant pressure filtration, on LRV [31]. Hongo-Hirasaki [32] observed a similar effect for the Planova 20N. The results obtained here indicate that aggregates similar in size to the virus particles can lead to broadening of the capture zone which increases the risk of virus particles passing through the membrane into the permeate; however, such broadening will only lead to decrease in LRV if the peak is close to the permeate edge of the membrane.

The movement of virus particle deeper into the membrane does not depend on the presence of similar sized aggregates alone. Besides the size of the aggregates, it will also depend on the permeate flux and filtration time. Lower fluxes due to fouling during constant pressure filtration and longer filtration times will lead to greater diffusional effects which will also permit virus particles to travel deeper into the membrane. By conducting experiments at constant flux, the effect of varying flux and filtration times have been eliminated in these studies. Finally, membrane properties such as asymmetry, porosity, and surface properties, as well as the feed conditions (level of product aggregation, pH, ionic strength, etc.) will affect the movement of virus particles in the membrane.

Table 4 and Table 5 indicate that under all conditions tested, the LRV was more than 4 logs. Further process pause appears to have little effect on LRV. While this may seem contrary to earlier studies [14,17,18] using fluorescent nanoparticles and bacteriophage, it is important to note that this is one of the first studies to investigate entrapment of MVM under industrially relevant conditions. Moreover, MVM was fixed on membrane surface immediately after virus filtration, eliminating the possibility of MVM diffusion after the filtration experiments. The membranes were also not overloaded in order to measure change in LRV. The results obtained here indicate that if the virus entrapment zone is far from the permeate side of the membrane (e.g., BioEX Figure 4c), the filter provides process robustness so that the peak shift and movement do not impact overall LRV.

## 4. Conclusions

Three commercially available hollow-fiber virus filtration modules were tested with two feed streams: MVM spiked into PBS buffer and 5 g L^−1^ BSA in PBS buffer spiked with MVM. Using fluorescently labeled MVM and LSCM, the location of virus entrapped was determined. The results indicate that a process pause leads to movement of the entrapment zone deeper into the membrane as well as some broadening of the entrapment zone. The presence of aggregate similar in size to MVM leads to a greater broadening of the entrapment zone. Both movement of the entrapment zone deeper into the membrane and broadening of the entrapment zone increases the possibility of lower LRV if virus particles are able to pass through the membrane. A more robust virus filter is one where the entrapment zone is far from the permeate side of the membrane. The results obtained here provide some of the first insights into movement of the zone of virus entrapment within the membrane under industrially relevant operating conditions

In this work the presence of BSA aggregates and the inclusion of a process pause did not lead to a change in the LRV (see Table 4 and Table 5). Here the membranes were not overloaded in order to measure difference in LRV. The results provide insights into the effects of aggregates similar in size to BSA and a process pause on virus entrapment in the membrane.

## Figures and Tables

**Table 1:** Details of three hollow-fiber virus filters.

Virus Filter	Filter Area (cm^2^)	Maximum Operating Pressure (MPa)	Membrane Polymer	Feed Volume (mL)
Planova™ S20N	10	0.216	Cuprammonium regenerated cellulose	1000
Planova™ 20N	10	0.098	Cuprammonium regenerated cellulose	1000
Planova™ BioEX	3.0	0.343	Hydrophilized polyvinylidene fluoride (PVDF)	300

**Table 2:** Summary of experiments conducted.

Virus Filter	Flux Measurements	Log Removal of Virus (LRV)	LSCM Imaging
20N	Buffer spiked with MVM (labeled and non labeled), data presented without process pause	Buffer spiked with MVM (labeled), data presented with and without process pause	Buffer spiked with MVM (labeled), data presented with and without process pause
	5 g L^−1^ BSA spiked with MVM (labeled and non labeled), data presented without process pause	5 g L^−1^ BSA spiked with MVM (labeled), data presented with and without process pause	5 g L^−1^ BSA spiked with, MVM (labeled), data presented with and without process pause
20N	Buffer spiked with MVM (labeled), data presented without process pause	Buffer spiked with MVM (labeled), data presented with and without process pause	Buffer spiked with MVM (labeled), data presented with and without process pause
	5 g L^−1^ BSA spiked with MVM (labeled), data presented with and without process pause	5 g L^−1^ BSA spiked with MVM (labeled), data presented with and without process pause	5 g L^−1^ BSA spiked with MVM (labeled), data presented with and without process pause
BioEX	Buffer spiked with MVM (labeled), data presented without process pause	Buffer spiked with MVM (labeled), data presented with and without process pause	Buffer spiked with MVM (labeled), data presented with and without process pause

**Table 3:** SEC data for BSA.

Feed Stream	Monomer (%)	Dimer (%)	Trimer (%)
BSA (5 g L^−1^, PBS buffer)	84.9	13.2	1.9

**Table 4:** MVM clearance for PBS buffer spiked with MVM S20N, 20N, and BioEX with (w/pp) and without process pause (w/o pp).

MVM	Planova™ S20N		Planova™ 20N		Planova™ BioEX	
	Feed (logTCID_50_ mL^−1^)	LRV	Feed (logTCID_50_ mL^−1^)	LRV	Feed (logTCID_50_ mL^−1^)	LRV
w/pp	5.17	≥5.54 ± 0.11	5.01	4.69 ± 0.13	4.83	≥5.21 ± 0.11
w/o pp	4.92	≥5.29 ± 0.13	5.00	4.69 ± 0.13	4.75	≥5.13 ± 0.14

**Table 5:** MVM clearance for 5 g L ^−1^ BSA in PBS buffer spiked with MVM for S20N and 20N with (w/pp) and without process pause (w/o pp).

5 g L^−1^ BSA with MVM	Planova™ S20N		Planova™ 20N	
	Feed Titer (logTCID_50_ mL^−1^)	LRV	Feed Titer (logTCID_50_ mL^−1^)	LRV
w/pp	4.83	≥5.20 ± 0.11	4.83	4.45 ± 0.13
w/o pp	4.83	≥5.20 ± 0.11	4.92	4.57 ± 0.11

**Figure A1:**
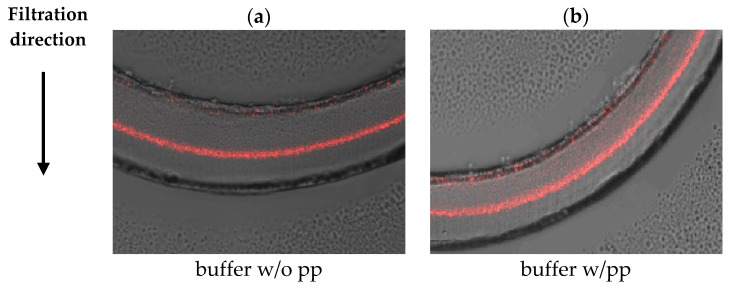
LSCM virus entrapment profiles for S20N, feed streams consisting of buffer spiked with labeled MVM. (**a**) Without process pause (w/o pp), (**b**) with process pause (w/pp).

**Figure A2:**
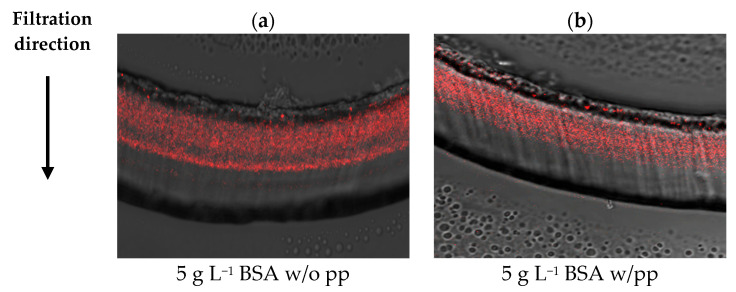
LSCM virus entrapment profiles for S20N, feed streams consisting of 5 g L^−1^ BSA spiked with labeled MVM. (**a**) Without process pause (w/o pp), (**b**) with process pause (w/pp).

**Figure A3:**
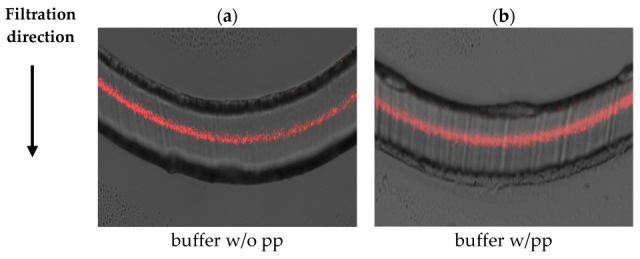
LSCM virus entrapment profiles for 20N, feed streams consisting of buffer spiked with labeled MVM. (**a**) without process pause (w/o pp) (**b**) with process pause (w/pp).

**Figure A4:**
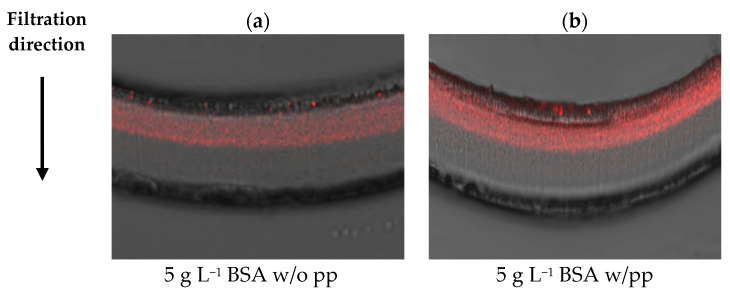
LSCM virus entrapment profiles for 20N, feed streams consisting of 5 g L^−1^ BSA spiked with labeled MVM. (**a**) Without process pause (w/o pp), (**b**) with process pause (w/pp).

**Figure A5:**
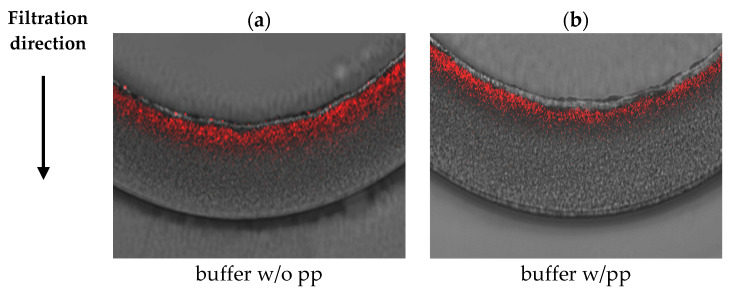
LSCM virus entrapment profiles for BioEX, feed streams consisting of buffer spiked with labeled MVM. (**a**) Without process pause (w/o pp), (**b**) with process pause (w/pp).

## Data Availability

The original contributions presented in this study are included in the article/Appendix A. Further inquiries can be directed to the corresponding authors.

## Appendix A

LSCM images of virus entrapment in the membranes.

**Table A1:** BSA recovery data for virus filtration spiked with labeled MVM without w/o pp) and with process pause (w/pp).

5 g/L BSA	Planova™ S20N	Planova™ 20N
w/o process pause	99.8% ± 0.08	99.7% ± 0.08
w/process pause	99.5% ± 0.01	99.5% ± 0.02
